# The Architecture of the TIR Domain Signalosome in the Toll-like Receptor-4 Signaling Pathway

**DOI:** 10.1038/srep13128

**Published:** 2015-08-21

**Authors:** Emine Guven-Maiorov, Ozlem Keskin, Attila Gursoy, Carter VanWaes, Zhong Chen, Chung-Jung Tsai, Ruth Nussinov

**Affiliations:** 1 Department of Chemical and Biological Engineering, Koc University, Istanbul, Turkey; 2 Center for Computational Biology and Bioinformatics, Koc University, Istanbul, Turkey; 3 Department of Computer Engineering, Koc University, Istanbul, Turkey; 4 Clinical Genomic Unit, Head and Neck Surgery Branch, National Institute on Deafness and Communication Disorders, NIH, Bethesda, MD 20892, USA; 5 Cancer and Inflammation Program, Leidos Biomedical Research, Inc. Frederick National Laboratory for Cancer Research, National Cancer Institute, Frederick, MD 21702, USA; 6 Sackler Inst. of Molecular Medicine, Department of Human Genetics and Molecular Medicine, Sackler School of Medicine, Tel Aviv University, Tel Aviv 69978, Israel

## Abstract

Activated Toll-like receptors (TLRs) cluster in lipid rafts and induce pro- and anti-tumor responses. The organization of the assembly is critical to the understanding of how these key receptors control major signaling pathways in the cell. Although several models for individual interactions were proposed, the entire TIR-domain signalosome architecture has not been worked out, possibly due to its complexity. We employ a powerful algorithm, crystal structures and experimental data to model the TLR4 and its cluster. The architecture that we obtain with 8 MyD88 molecules provides the structural basis for the MyD88-templated myddosome helical assembly and receptor clustering; it also provides clues to pro- and anti-inflammatory signaling pathways branching at the signalosome level to Mal/MyD88 and TRAM/TRIF pro- and anti-inflammatory pathways. The assembly of MyD88 death domain (DD) with TRAF3 (anti-viral/anti-inflammatory) and TRAF6 (pro-inflammatory) suggest that TRAF3/TRAF6 binding sites on MyD88 DD partially overlap, as do IRAK4 and FADD. Significantly, the organization illuminates mechanisms of oncogenic mutations, demonstrates that almost all TLR4 parallel pathways are competitive and clarifies decisions at pathway branching points. The architectures are compatible with the currently-available experimental data and provide compelling insights into signaling in cancer and inflammation pathways.

## Highlights

- The signalosome architecture provides the structural basis for TIR-domain signaling
- The TIR domain signalosome illuminates receptor clustering upon stimulation
- Almost all parallel pathways of TLR4 signaling are competitive
- Structural details of interactions reveal the mechanisms of oncogenic mutations

**Figure 1:**
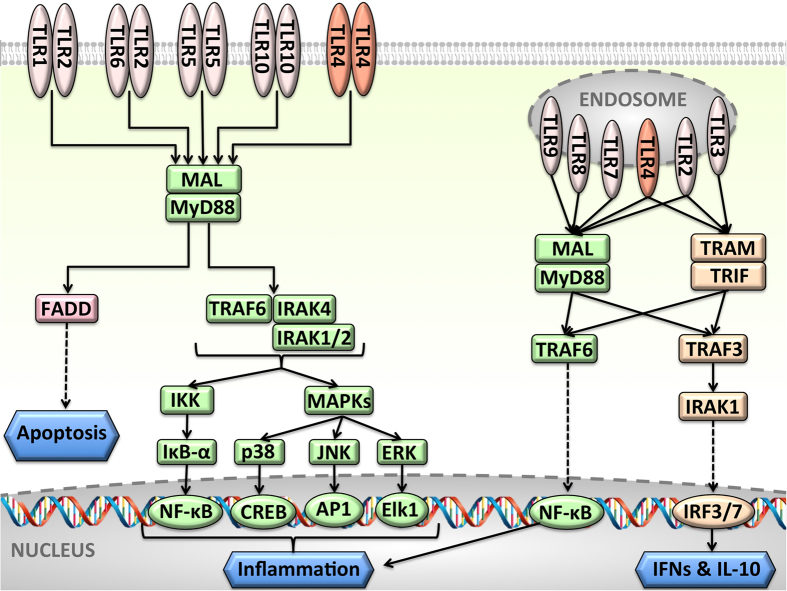
Toll-like receptor pathway (adapted from literature^12^,^15^,^19^), in traditional node-and-edge representation, where nodes represent proteins and edges represent interactions between proteins. TLR pathway is complicated and has many branches. Stimulation of TLRs propagate the signal through two parallel paths: MyD88-dependent path (green), which leads to production of pro-inflammatory cytokines, and TRIF-dependent path (orange), which gives rise to transcription of antiviral proteins—interferons—and anti-inflammatory cytokine IL-10. MyD88-mediated pathway also has three branches, namely TRAF6- (green), TRAF3- (orange) (downstream of endosomal TLRs), and FADD-dependent (pink) downstream pathways. For space limitation, we showed TLRs on endosomal membrane as monomers, but they also dimerize upon stimulation.

Toll-like receptors (TLRs) orchestrate the innate and adaptive immune systems^1^. The TLR pathway (Fig. 1) plays critical roles in almost every phase of tumor development^2^. Two opposing roles are attributed to TLRs: anti-tumor and pro-tumor actions^3^. TLR-induced inflammation promotes cancer via proliferative and anti-apoptotic factors^4^. TLRs form homo- or hetero-dimers and their cytoplasmic Toll/IL-1R homology (TIR) domains associate with TIR domain-containing adaptor molecules to stimulate signaling^5^. They have six adaptor proteins, Myeloid differentiation factor 88 (MyD88), MyD88 adaptor-like (Mal, also known as TIRAP)^6^, TIR domain containing adaptor inducing interferon-β (TRIF, also known as TICAM-1)^7^, TRIF-related adaptor molecule (TRAM, also known as TICAM-2)^8^, sterile α and heat-armadillo motifs (SARM)^9^, and B-cell adaptor for PI3K (BCAP)^10^. TLR signaling induces expression of pro-inflammatory cytokines, interferons (IFNs) and interleukin-10 (IL-10, an anti-inflammatory cytokine). While IFN production suppresses cancer, pro-inflammatory cytokines promote it^11^. Upon stimulation, TLRs cluster in lipid rafts^12^,^13^. In their MyD88-dependent pathway, the TLR TIR domains associate with TIR domains of MyD88 and Mal proteins. MyD88 TIR domain is connected through a long linker to its DD. Through its DD, MyD88 can initiate three downstream pathways. In the first, pro-survival inflammatory pathway, it recruits serine/threonine kinases IRAKs (Interleukin-1 receptor-associated kinases) to stimulate the TNF receptor-associated factor 6 (TRAF6), IKK complex and MAPKs, (e.g. ERK, JNK, and p38^14^) and transcription factors NF-κB, AP-1, and CREB^15^,^16^, which ultimately result in transcription of pro-inflammatory cytokines, such as tumor necrosis factor-alpha (TNF-α), and IL-1β^17^. In the second path, MyD88 DD binds to TRAF3 instead of TRAF6 (only TLRs on endosomal membranes recruit TRAF3^18^)^19^. TRAF3 is a negative regulator of TLR- and TNFR-mediated MAPK activation and has to be degraded for MAPK stimulation^19^. Instead of activating NF-κB, it activates interferon regulatory factors (IRFs)^20^. In the third death path, MyD88 DD associates with FADD (Fas-associated death domain) protein, which leads to apoptosis. In the TRIF-dependent pathway, IRFs dimerize and get activated, producing IFNs. Whether Mal and TRAM bind to TLR4 competitively using the same interaction surface has been unknown^21^, but several studies pointed out that they do^18^,^21^,^22^. This is important since it could explain the outcome of inflammation/cancer-related aberrations or mutations on the Mal and TRAM binding surfaces, or overexpression of either of these. It was suggested that upon engagement of TLR4 with its cognate ligand lipopolysaccharide (LPS), these two pathways are activated sequentially: first the MyD88-dependent and then the TRIF-dependent^7^,^12^,^18^.

At almost all levels of TLR signaling, proteins oligomerize to form large multimolecular assemblies^23^,^24^. TIR-domain containing adaptors form the TIR-domain signalosome; DD-containing proteins form the myddosome^13^; and TRAF6 forms an infinite network by trimerization of their TRAF-C domains and dimerization of their RING-domains^25^. Oligomerization of proteins facilitates execution of cellular functions by amplifying signals and allowing more efficient enzymatic reactions^26^. In support of this, it has been demonstrated that induced TLR4 clustering activates TLR4 even in the absence of its ligand LPS^24^.

### TIR-domain signalosome assembly

TLR1, TLR2, TLR10, Mal, MyD88, TRAM, TRIF, and IL-1RAPL have resolved structures of their TIR-domains. Among these, TLR10 (PDB_ID: 2j67), C713S mutant TLR2 (PDB_ID: 1o77), and IL-1RAPL (PDB_ID: 1t3g) are in homodimer form. Each TIR domain is composed of five central β-strands (A-through-E) and six surrounding α-helices (A-through-E)^27^. The loops that connect helices and strands are named by the elements that they link. Although the overall structure is similar across TIR domains, their loops vary^28^. TIR-domain containing proteins associate through TIR-TIR interactions and form multimeric signalosomes. Several models were proposed and almost all point to the importance of the BB-loop^6^,^8^,^21^,^27^,^29^,^30^,^31^. These studies identify interface residues by mutagenesis. However, there are some contradictions among the studies relating to interface residues that are involved in TIR-TIR interactions. For instance, the C747S mutation is said to inhibit TLR4 homodimerization^21^, whereas other studies suggested that blockage of C747 by a small molecule TAK-242 (resatorvid) inhibits TLR4 signaling not because it interferes with TLR4 dimerization, but because it abolishes TLR4-Mal and TLR4-TRAM interactions^29^. We draw two conclusions from these findings: some mutations may be allosteric and are not necessarily on the interface, or there is more than one binding mode.

**Figure 2:**
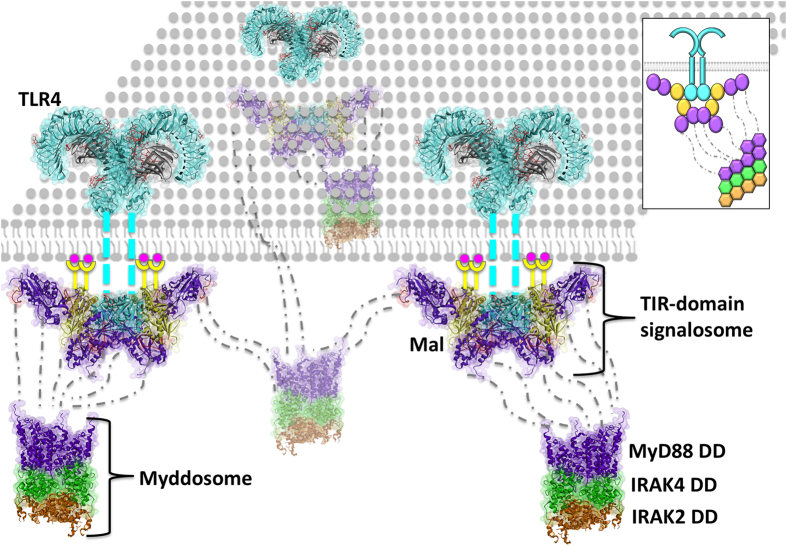
3D schematic view of TIR-domain signalosome, myddosome and TLR clustering. It is known that TLRs cluster on lipid rafts, but they cannot tetramerize due to the steric hindrance of their ectodomains^26^,^34^. Oligomerization of the downstream proteins may hold TLRs together. Here, all TIR domains are in dimer form, TLR4, Mal, and MyD88. A TLR4-dimer recruits two Mal-dimers, which in turn recruit four MyD88-dimers. In the myddosome complexes, there are six MyD88 molecules, four IRAK4 and four IRAK2. The box at the upper right corner shows the cartoon version of the model. The PDB_ID of the myddosome complex is 3mop: MyD88 death domains 3mopBCDE, IRAK4 death domains 3mopGHIJ, IRAK2 death domains 3mopKLMN. Pink circles are PI(4,5)P2, which are enriched in lipid rafts and N-terminal region of Mal associates with it.

Mutagenesis studies identified interface residues and led to structural models of some of the binary interactions of TIR-domain signalosome^6^,^8^,^21^,^27^,^29^,^30^,^31^ but not of the entire complex. Here, we model the MyD88- and TRIF-dependent signalosomes by exploiting the powerful PRISM algorithm^32^,^33^. The architecture that we obtain provides the structural basis for TLR clustering through formation of a TIR-domain signalosome with 8 MyD88 molecules and a helical myddosome crystal structure with 6 MyD88 (Fig. 2). Our binary interactions are compatible with available experimental data. Significantly, our results reveal how regulation at key anti- and pro-inflammatory signaling checkpoints takes place, providing insight into TLR and MyD88 signaling decisions.

## Results and Discussion

### Multimeric TIR domain signalosome assembly and TLR clustering

TLRs’ clustering is crucial for efficient signaling, but they cannot form clusters through tetramerization or higher order oligomerization due to steric hindrance of their ectodomains^26^,^34^. Instead, oligomerization of downstream proteins may hold TLRs in close proximity via a linked network mesh. Here, we built MyD88- and TRIF-dependent TIR domain signalosomes, comprising TLR4/Mal/MyD88 or TLR4/TRAM/TRIF. The signalosome models are meshed through the myddosomes whose crystal structure is available (Fig. 2). The linker region (45 residue-long) of MyD88 between its TIR and DD is essential for the TLR clustering. A splice variant of MyD88, so called MyD88s (short MyD88), lacking the interdomain linker region has been shown to inhibit NF-κB activation^35^ and this outcome is attributed to its inability to recruit IRAK4, which is necessary for nucleation of the myddosome assembly. In support of this, our model suggests that without myddosome formation, TLRs cannot cluster and no signal is relayed to downstream effectors.

In our MyD88-dependent signalosome, all TIR domains are in dimer form: a TLR4-dimer recruits two Mal-dimers, which in turn recruit four MyD88-dimers. Different signalosome schemes, with varying stoichiometries of Mal and MyD88 were proposed before: some of them show 2 Mal and 2 MyD88 molecules per TLR4 dimer in the signalosome^36^,^37^, whereas others include 2 Mal and 4 MyD88 (2 dimers)^38^. However, studies clearly revealed that both Mal^27^ and MyD88^39^,^40^ should be in dimeric form to assist the signaling by serving as a binding platform. Different signalosome schemes, with varying stoichiometries of Mal and MyD88 will give rise to different mesh-like structure scenarios. Below we outline the step-by-step construction of TIR domain signalosomes.

### TLR4 Dimerization

**Figure 3:**
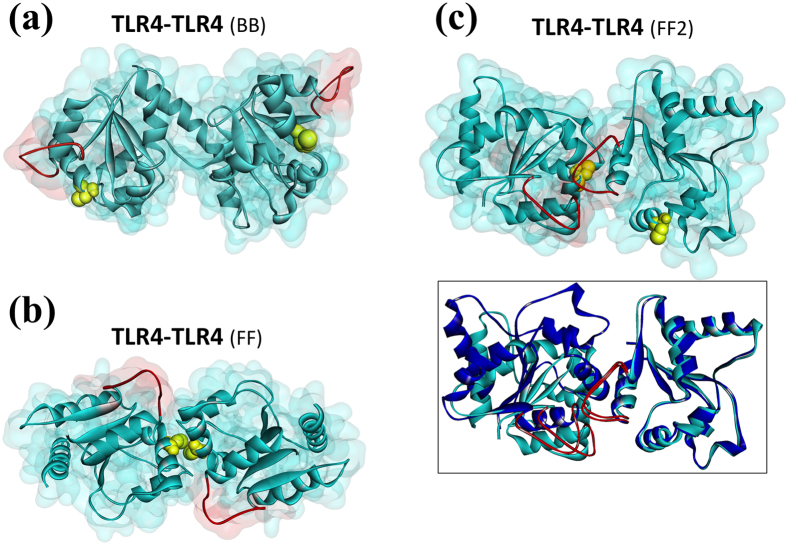
TLR4 homodimer models. (**a**) Back-to-back TLR4 dimer (BB). (**b**) Face-to-face TLR4 dimer (FF), which is very similar a previously suggested TLR4-dimer model^21^ and crystal structure of the dimeric TIR domain of IL-1RAPL (1t3g.pdb)^42^. (**c**) Another face-to-face dimer (FF2) in which BB-loops are in very close proximity. The box in the lower left corner shows the structural alignment of this TLR4-homodimer model with the one that is obtained by superimposition of TLR4 with TLR2 homodimer crystal structure (1o77_CD), (146 of 276 residues with 0.73 RMSD by multiport). Cyan color is TLR4 TIR domain, red-labeled regions are BB-loops, and yellow spheres are C747 residues on each TLR4 TIR domain, which are suggested to be involved in the interface. The dark blue dimer in the box is TLR4-dimer, which is obtained by superimposition with TLR2 dimer (1o77_CD).

Upon stimulation, TLRs form homo- or hetero-dimers with their Leucine Rich Repeats (LRRs) and TIR domains^22^. The structure of the TLR4 TIR-domain has not yet been resolved. We built its model by the I-TASSER server (residues 672-818)^41^. The crystal structure of the TLR1 TIR domain (PDB_ID: 1fyv) was used as the template. The model has 1.29 Å RMSD with TIR domains of other TLRs over 111 residues and other TLRs have 1.22 Å RMSD over 112 residues with each other. Several models have been proposed for the TLR4-TLR4 interaction based on mutagenesis^21^,^22^,^29^ with disagreements among these with respect to interface residues^21^,^29^. Such diverse findings for the interface region may suggest different binding modes for TLR4 dimerization. Also, the presence of other partners, like Mal, may change the TLR4 binding mode preference. In line with this idea, we found three different TLR4-homodimer organizations (Fig. 3). Details of the interactions are in Table S1.

In the first potential TLR4-homodimer model (Fig. 3a), BB-loops face opposite directions (back-to-back dimer, BB), contrary to what has been suggested before^21^. The second with BB-loops facing each other (face-to-face, FF) (Fig. 3b) is very similar to a previously proposed TLR4-homodimer^21^ and a crystal structure of the dimeric TIR domain of IL-1RAPL (1t3g.pdb)^42^. In addition, the C747 residues that have been suggested to be at the interface^21^ are very close to each other. FF interface might be the major interface for downstream signaling. The third model shown in Fig. 3c is very similar to TLR2-homodimer crystal structure (1o77_CD), in which the BB-loops are in close proximity (face-to-face-2, FF2). Although this structure is very similar to a crystal packing TLR2-dimer it is not plausible with the downstream TIR-domain interactions, which have the mutation-indicated interface residues at the correct sites: meaning that that this interface has steric clashes with downstream interactions. The homodimer captured in the crystal may not be the physiological conformation. We thus built the TIR-domain signalosome complexes for the BB and FF models.

### Mal Dimerization

Mal has a TIR-domain and a small amino terminal localization domain, through which it can interact with phospholipids, particularly phosphatidylinositol-4,5 bisphosphate (PI(4,5)P2) that is enriched in lipid rafts^37^. Mal is a homodimer *in vivo*^6^. Recently, it was suggested that Mal dimerization facilitates its interactions with MyD88 and TLR4 by forming a binding platform^27^. Unlike other TIR-domains, Mal TIR-domain lacks a BB-loop, but has an extraordinarily extended AB-loop. BB-loops of other TIR domains correspond to a part of Mal’s AB-loop^6^,^27^. In Mal crystal structures (4fz5, 2y92, 3ub2, 3ub3, 3ub4, 4lqd), many of Mal’s residues (21 residues) are missing. The asymmetric unit of the crystal (3ub2.pdb) displays a symmetrical back-to-back Mal-dimer with the AB-loops facing the opposite direction^29^, which has been suggested to be the physiological state^6^. We obtained a Mal-homodimer organization, which is very similar to the unit cell Mal-dimer (Supplementary Figure S1). Residues P155, W156, K158 and E190 that mutagenesis suggested to be involved in the interface of Mal-homodimer^6^,^27^ are in the interface of our Mal-homodimer. In addition, the N-termini of the both monomers face the same direction, such that both could attach to the PI(4,5)P2 in the membrane.

### TLR4-Mal Interaction

**Figure 4:**
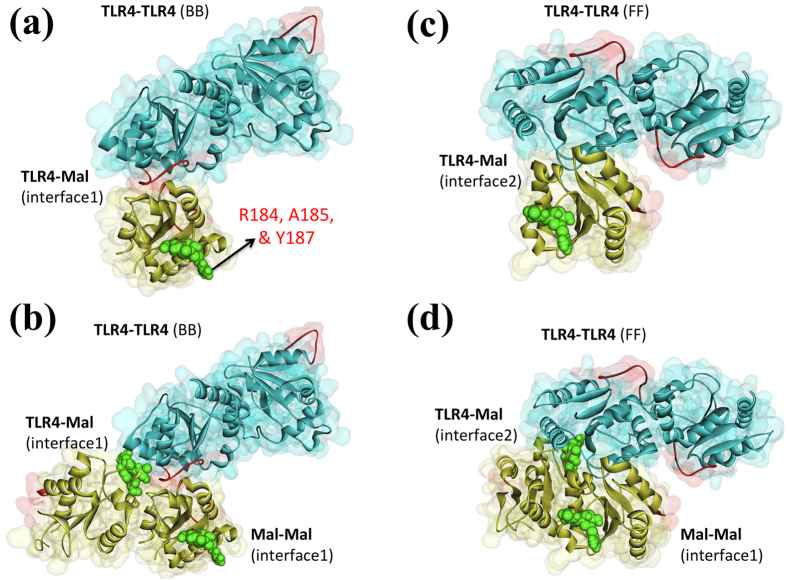
Interaction models of Mal-monomer (**a**,**c**) and Mal-dimer (**b**,**d**) with BB and FF TLR4-homodimer models. Yellow protein is Mal and green spheres show the proposed interface residues of Mal (R184, A185, Y187)^27^, none of which are at the correct site in the monomeric-Mal-TLR interaction model. However, if dimerization of Mal is also taken into account, it is seen that both monomers are in contact with TLR4, one of which has the interface residue at the correct site.

Like the TLR4-homodimer, we predicted several architectures for the TLR4-Mal interaction. The two TLR4-dimers (BB and FF) use different interfaces to interact with Mal TIR-domain, suggesting that distinct TLR4-Mal architectures are possible (Fig. 4a,c). However, the previously suggested interface residues of Mal (R184, A185, & Y187)^27^ are not at the correct site. When we superimpose the Mal-homodimer on the TLR4-Mal complexes, we observed that both Mal monomers are in contact with TLR4 and one of the Mal monomers has the proposed interface residues at the correct site (Fig. 4b,d). This underscores the importance of higher order oligomerization modes while deciphering signaling pathways.

### Mal-MyD88 Interaction

**Figure 5:**
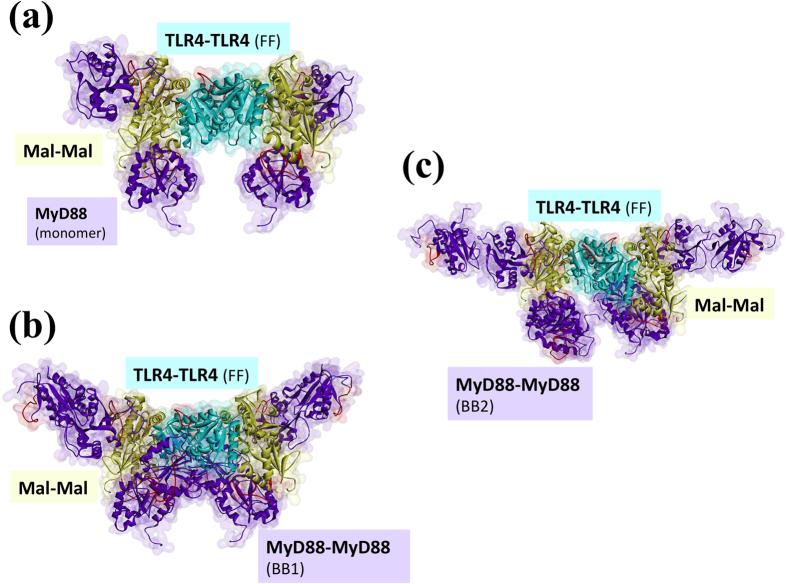
Possible TIR domain signalosome models for FF TLR4-dimer. (**a**) Interaction model of monomeric-MyD88 with TLR4 and Mal dimers. (**b**,**c**) MyD88-dependent TIR-domain signalosome models for FF TLR4-dimer. All proteins are in dimer form, including TLR4, Mal, and MyD88. It is known that dimeric MyD88 has higher affinity to stimulated TLRs due to their extended interfaces. In line with this, models (**b**,**c**) show that the second MyD88 of the MyD88-dimer is very close to TLR4. Especially in part-c, one of the MyD88 molecules in the dimer is bound to Mal, and the other is bound to TLR4. We obtained these complexes by superimposition of the binary interaction models of TLR4-TLR4, TLR4-Mal, Mal-Mal, Mal-MyD88 and MyD88-MyD88.

Mal serves as a bridge between TLR4 and MyD88. Mutational analysis indicated that MyD88 R196 and R288 are at the Mal-MyD88 interface^6^,^30^. However, these two residues fall on opposite sides of MyD88, indicating that there is more than one bound conformation for the Mal-MyD88. Among several Mal-MyD88 architectures, only one features TLR4- and Mal-homodimers with R196 at the interface (Supplementary Figure S1), when superimposed with TLR4-Mal interactions. Figure 5a and Supplementary Fig. S2 show the signalosomes of TLR4-Mal-MyD88 for the two possible TLR4-homodimers, FF and BB, respectively. As in TLR4-Mal interaction where the suggested interface residues are at the correct site only when Mal is in dimer form, R288 of MyD88 is in contact with TLR4 only if MyD88 dimerizes (Fig. 5b and Supplementary Figure S3).

### MyD88 Dimerization

In order to form the myddosome, comprising MyD88, IRAK4 and IRAK2/IRAK1, MyD88 molecules should dimerize and oligomerize. Although variable stoichiometries have been observed (8:4:4, 7:4:4, 6:4:4)^13^,^24^ a more favored myddosome organization should have six MyD88 molecules^13^. The crystal structure of the helical myddosome involves six MyD88 DDs, four IRAK2 and four IRAK4 DDs^13^. The myddosome complex has four layers: layer-1 has four IRAK2 DDs, layer-2 has four IRAK4 DDs, layer-3 has four MyD88 DDs, and layer-4 has the next two MyD88 DDs. MyD88 dimerization through both its TIR and DD is necessary for assembly into myddosome^39^. Inhibition of the dimerization of its TIR domain by peptidomimetic compounds blocks the assembly of the myddosome^40^. Therefore, we include MyD88-dimer models in the TIR-domain signalosome (Fig. 5b,c, Supplementary Figure S2).

An earlier study suggested more than one binding mode for TIR domain MyD88-dimerization and formation of multivalent aggregates^34^. In line with this, we found four MyD88-dimer organizations (Supplementary Figure S4), two FF and two BB. However, only BB dimers (Supplementary Figure S4) are possible for the TIR domain signalosome TLR4-Mal-MyD88 interactions. Some viruses employ TIR domain containing proteins (Tcp) to suppress TLR-mediated host immune response^34^. The crystal structure of a TIR domain dimer of TcpB of *Brucella* (4lqc.pdb) is also BB^43^. This may support the BB MyD88-dimer architectures as being feasible with the whole TIR-domain signalosome.

Figure 5b,c, and supplementary Fig. S2 display the superimposition of the BB MyD88-dimers with two possible TLR4-Mal-MyD88 interaction modes. MyD88-dimers have higher affinity for stimulated TLRs than monomeric MyD88 because of the extended interfaces of dimeric MyD88 TIR domains^34^. Remarkably, with our preferred (FF) TLR4-dimer model, if monomeric MyD88 is recruited to the TIR-domain signalosome, this MyD88 is away from TLR4 (Fig. 5a), but if MyD88-dimers bind to the TIR-domain signalosome, two of the MyD88 molecules get very close to the TLR4 dimers (Fig. 5b,c). In particular, in the assembly shown in Fig. 5b, one MyD88 molecule of the MyD88-dimer is bound to Mal and the other to TLR4. Supplementary Fig. S3 provides the details. MyD88-dimer may indeed have higher affinity for activated TLR4, because it is in contact with both Mal and TLR4 itself. Although the first MyD88 in supplementary Fig. S3 does not have any contacting residues with TLR4, the second MyD88 has 21 interacting residues according to the HotPoint server^44^. Consequently, a TLR4-dimer recruits two Mal-dimers and four MyD88-dimers. Although the stoichiometry of MyD88 in the myddosome complex was determined, there are no such data for the signalosome. Nevertheless, it was suggested that as long as MyD88 TIR domains are in a dimer form, it is not that critical how many MyD88 molecules are in the signalosome^34^. Figure 2 provides an overview of the TLR/Mal/MyD88 signalosome, myddosome and TLR clustering.

### TRAM Dimerization

Similar to Mal, which is a bridging adaptor and associates with phospholipids in the membrane, TRAM is also a bridging adaptor and is attached to the membrane via its myristoyl group^12^. TRAM homodimerization is crucial for recruitment of TRIF^8^. In our TRAM-dimer architectures, residue H117 is at the interface, as suggested earlier^8^ (Supplementary Figure S5). This dimer is similar to TLR10 homodimer in the crystal structure (2j67_AB) as shown in the box in supplementary Fig. S5.

### TLR4-TRAM Interaction

TRAM links TLRs to TRIF, just like Mal connecting TLRs to MyD88. The C747 residue of TLR4 was shown to be involved in the TLR4-TRAM interface^6^,^29^. However, this residue was also shown to be at the TLR4-homodimerization interface^21^, suggesting different binding conformations. Supporting this assumption, we found different architectures for the TLR4-TRAM interaction. For each TLR4-homodimer (FF and BB), only one TLR4-TRAM which does not interfere with the TLR4-homodimer is possible (Supplementary Figure S5). None of them have the C747 residue of TLR4 at the TLR4-TRAM interface.

**Figure 6:**
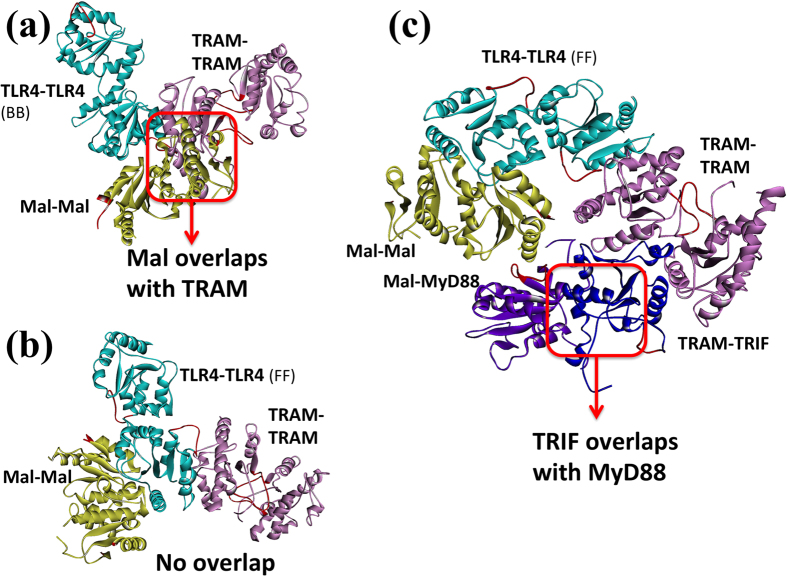
MyD88- and TRIF- dependent downstream TLR pathways are mutually exclusive. (**a**) TRAM-homodimer has a steric clash with Mal-homodimer when superimposed to BB TLR4-homodimer model, and thus they are mutually exclusive: either Mal or TRAM homodimers can bind to TLR4 at any time. TRAM and Mal interactions are mutually exclusive in BB TLR4-homodimers and this is in line with the findings of several studies^18^,^21^,^22^. This indicates that MyD88-dependent pro-inflammatory and TRIF-dependent anti-inflammatory pathways are competitive. (**b**) TRAM-homodimer does not overlap with Mal-homodimer when superimposed to FF TLR4-homodimer model. (**c**) MyD88 overlaps with TRIF on TLR4: the FF TLR4-homodimer model has steric clashes of MyD88 and TRIF when superimposed Mal-MyD88 and TRAM-TRIF. Red box indicates the location of steric clash.

TRAM homodimer formation is required for TRIF recruitment. Supplementary Fig. S5 displays the TLR4-homodimers that are bound to TRAM-homodimers. Supplementary Fig. S6 shows TLR4-TRAM-TRIF interactions with two TLR4-homodimers. When we superimpose the Mal-homodimers onto these TLR4-TRAM, we observe that TRAM and Mal interactions are mutually exclusive in the BB TLR4-homodimer since they have overlapping binding sites on TLR4 (Fig. 6a), but not with the FF TLR4 dimer (Fig. 6b). Proteins that bind to identical or overlapping interfaces on a protein will have a steric clash and thus cannot bind simultaneously^45^.

### TRAM-TRIF Interaction

TRAM acts as a scaffold bringing TLRs and TRIF together. This assembly is a key upstream branching step in the interferon and anti-inflammatory pathway (Fig. 1). Monomeric TRIF is able to bind to TRAM homodimers^8^, suggesting that there is no need for TRIF dimerization. The residues that are proposed to be at the TRAM-TRIF interface include Q512, I519 (QI-site), R522, K523 (RK-site) of TRIF and T155, S156 (TS-site), E87, D88, D89 (EDD-site) of TRAM^8^. The TRAM-TRIF interaction model is shown in supplementary Fig. S6 and it has QI, RK, and EDD-sites at the interface, but not the TS-site. When the MyD88-mediated signalosome is superimposed on the TRIF-mediated signalosome based on the FF TLR4 dimer (the major TLR4-dimer for signaling), MyD88 and TRIF present a steric clash (Fig. 6c). Both BB and FF TLR4 dimers have steric hindrance when MyD88- and TRIF-dependent signalosomes are superimposed. This is in line with the findings of several studies^18^,^21^,^22^. Importantly, this indicates that MyD88-dependent pro-inflammatory and TRIF-dependent anti-inflammatory pathways are competitive and thus restrict the activation of one another. This may offer a means of regulation to the TLR signaling. However, it is important to note that these parallel paths only switch the function: while negatively regulating one path, they positively regulate others downstream of TLRs.

### Interactions of Downstream Players with DD of MyD88

**Figure 7:**
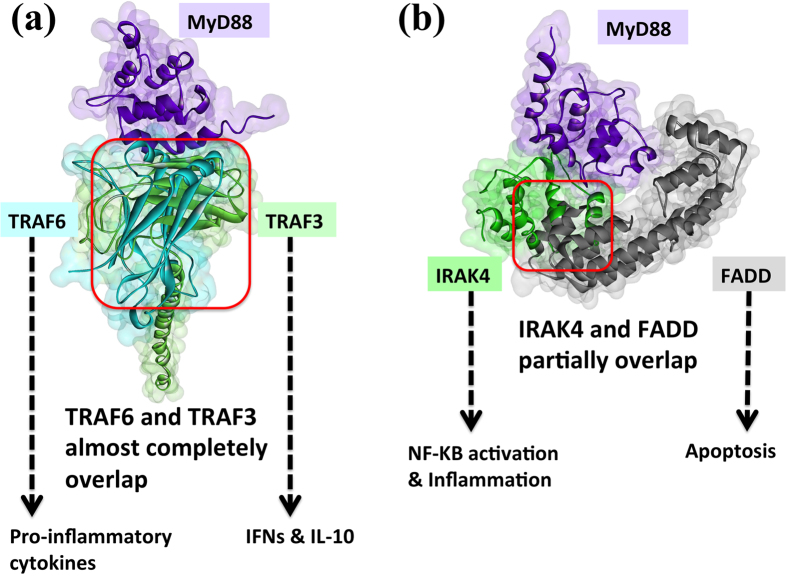
MyD88 interaction models with the downstream orchestrators reveal that the three parallel downstream paths are competitive. (**a**) TRAF6 (1lb5:A) and TRAF3 (1fll:A) binds to almost completely overlapping interfaces on MyD88 DD (3mop:F), thus they are mutually exclusive. (**b**) IRAK4 (3mop:J) and FADD (2gf5:A) bind to overlapping interfaces on MyD88 DD (3mop:F), thus they compete to bind to MyD88. MyD88-IRAK4 interaction is not PRISM prediction, where the crystal structure of the complex is available (3mop:FJ). Red box indicates the location of steric clash.

Clustering of MyD88 DDs initiates the oligomerization of the myddosome complex. Besides IRAK4 and IRAK2, MyD88 DDs also associate with TRAF6^46^, TRAF3^11^, and FADD^47^,^48^. Schematic representations suggested that downstream proteins, like TRAF6, interact with IRAK1/2, but not with MyD88^15^,^26^. However, a recent study revealed that there is also a direct interaction between MyD88 and TRAF6 and abrogation of this interaction inhibits NF-κB activation^46^. In line with this, we observed that TRAF6 prefers to bind to MyD88 when the whole myddosome is given as a target instead of monomeric MyD88 or IRAK2. That is, although there are favorable interactions of TRAF6 with both monomeric MyD88 and monomeric IRAK2 (Supplementary Figure S7), when the whole myddosome is taken into consideration, TRAF6 selects MyD88. As we stated before, higher order oligomerization is important for function and should be considered in modeling. We select the myddosome-TRAF6 interaction, with TRAF6 bound to MyD88, instead of the monomeric IRAK2-TRAF6. Figure 7 illustrates the interaction of TRAF6 with MyD88; the detailed supplementary Fig. S8 shows that the myddosome-TRAF6 organization in which the TRAF-C domain of TRAF6 is in contact with DDs of two MyD88 molecules (two layers of MyD88), one interaction is major, with hotspots and the other further stabilizing the complex. The interface is similar to the concave TRAF binding site with peptides as observed in TRAF6-CD40 (1lb6.pdb)^49^, TRAF2-TRADD (1f3v.pdb)^50^, and TRAF2-OX40 (1d0a.pdb)^51^. The TRAF-C region of TRAF6 needs to trimerize to function^25^ and TRAF6 trimerization is possible with this myddosome-TRAF6 architecture (Supplementary Figure S8). Previous studies showed that TRAF3 also associates with MyD88-IRAK4-IRAK1 complex^52^. Similar to TRAF6, TRAF3 also associates with two MyD88 proteins (two layers of MyD88) in the myddosome (Supplementary Figure S8). MyD88 binds to the concave site on the TRAF-C region of TRAF3, as previously observed in other interactions such as TRAF3-CD40 (1fll.pdb)^53^, TRAF3-BAFFR (2gkw.pdb)^54^, TRAF3-LMP1 (1zms.pdb)^55^, and TRAF3-Cardif (4ghu.pdb)^56^.

Endosomal TLRs can signal through both TRAF3 and TRAF6. TRAF6 signaling activates the classical NF-κB pathway, leading to expression of pro-inflammatory cytokines^52^. On the other hand, TRAF3, a negative regulator of MAPKs and the alternative NF-κB pathway^19^ induces production of anti-inflammatory cytokine IL-10^20^,^52^ (Fig. 1). We observed that TRAF6 and TRAF3 bind to almost completely overlapping interfaces on MyD88 (Fig. 7a). This may assign a new regulatory role for TRAF3 in TLR signaling: the presence of TRAF3 restricts the activation of NF-κB and give rise to production of IFNs and IL-10.

FADD is another protein interacting with MyD88 DD^46^. It is a negative regulator of TLR signaling by suppressing LPS-induced NF-κB activation through possible competition with IRAK4 for binding to MyD88^47^,^48^. Considering the MyD88-FADD organization and the MyD88-IRAK4 crystal structure, MyD88 exploits (partially) overlapping surfaces to interact with IRAK4 and FADD (Fig. 7b). This organization explains why FADD hinders IRAK4 binding to MyD88 and thus myddosome assembly. In addition, Fas activation promotes TLR signaling and chronic inflammation^47^. If Fas and TLRs are activated simultaneously, activated Fas sequesters FADD and liberates MyD88, allowing constitutive inflammation^48^.

Remarkably, the C27* nonsense mutation on FADD protein, which is clinically observed in lung squamous carcinoma with 0.21 frequency (according to the TCGA data)^57^, abolishes the MyD88-FADD interaction (Supplementary Figure S9) can be explained by this architecture. Since truncated FADD can no longer occupy the MyD88 binding site, TRAF6 and IRAK4 are able to bind. This activates MAPKs, which induce production of pro-inflammatory cytokines, and prevents induction of apoptosis. This may clarify how the C27* mutation on FADD contributes to initiation or progression of tumor. Another mutation, the R34H missense mutation on FADD, observed in stomach adenocarcinoma, falls just next to the interface region (Supplementary Figure S9). Our model suggests that this mutation decreases the affinity of FADD to MyD88 and may block TLR-mediated apoptosis.

**Figure 8:**
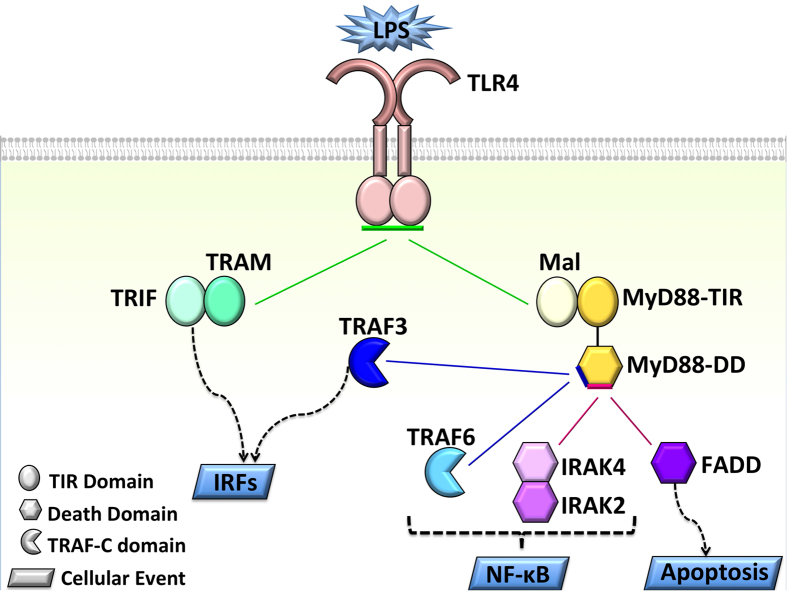
Parallel downstream pathways of TLRs, which lead to distinct outcomes, are mutually exclusive. Green arrows shows that TRIF- and MyD88-dependent paths cannot be activated simultaneously due to shared binding sites on TLR4-dimer or steric hindrance. Blue arrows demonstrate that TRAF6 and TRAF3 bind to overlapping interfaces on MyD88 DD (downstream of endosomal TLRs). Pink arrows shows that IRAK4 and FADD will have steric clash when they bind to MyD88 at the same time. The three branches of TLR pathway, namely pro-inflammatory, interferon and anti-inflammatory, and apoptotic paths are mutually exclusive.

Taken together, the TLR4 architectures indicate that all TLR’s parallel downstream pathways are competitive (Fig. 8). MyD88- and TRIF-dependent pathways downstream of a single TLR4-dimer cannot be activated simultaneously due to shared binding site. If MyD88 is recruited to the activated TLRs, it uses its TIR domain to interact with the bridging adaptor protein Mal/TIRAP and its DD to interact with the other downstream partners, IRAKs, TRAF6, TRAF3, and FADD. These trigger three alternative parallel pathways and lead to distinct/opposing outcomes.

To conclude, considerable effort has been invested in the quest for the entire TIR signalosome assembly, including its clustered architectures. This problem is significant since TLR activation involves clustering and signalosome formation. As we show here, the architectures may clarify TLR4 physiological signaling control and how it can go wrong in disease. Here, we present the two, MyD88-dependent and TRIF-dependent TIR-domain signalosome assemblies. We exploit experimental mutational data in every step in the construction. TLR activation through its ectodomain dimerization can elicit the proinflammatory, anti-viral and anti-inflammatory, and apoptosis pathways. Binding to Mal/MyD88 or TRAM/TRIF is the step making the first cellular decision. Our results suggest that steric hindrance of Mal and TRAM in a TLR/TRAM/TRIF assembly and Mal/MyD88 and TRIF leads to competitive binding to TLR’s TIR domain. Our results further reveal that parallel downstream pathways with opposing consequences are competitive at almost each branching point of the TLR pathway, beyond Mal and TRAM recruitment. TRAF6, TRAF3, and FADD, whose recruitment results in pro-inflammatory, anti-inflammatory, and death pathways respectively, present similar scenarios. Our signalosome architectures with 8 MyD88 molecules are important, since they provide the basis for obtaining an insight into how TLR4 clusters. The 8 MyD88 molecules connect into multiple 6 MyD88 molecules myddosome helical signaling units, and unveil downstream oligomerization clusters formed by stimulated TLRs. We speculate that the long MyD88 linker is critical for TLR clustering, and could be its raison d'être. The clusters, and their multivalent network, particularly through TRAFs^26^, allow efficient signaling, even with reduced TLR concentration since it enables signal amplification. Finally, our models can help explain the mode of action of relevant human mutations^58^,^59^.

## Methods

### Modeling Protein-Protein Interactions and Construction of the Structural TLR Network

We obtained the upstream TIR-domain interactions and downstream DD interactions based on binary interactions of proteins predicted by PRISM^32^,^33^. PRISM is a template-based algorithm. It utilizes prior interface knowledge of known 3D structures of protein-protein interaction (PPI) complexes and predicts structural interactions of target proteins. If the experimental 3D structure of the target protein is missing from the PDB, we build models of that protein by exploiting the I-TASSER server^41^. For a pair of target proteins, PRISM may generate more than one model. Therefore, it is possible to build numerous distinct oligomeric complexes. However, we think that the most stable complex is the one that is supported by the experiments. In order to determine which model is more stable and physiologically relevant, we crosscheck the interface residues of our models with available mutational/biochemical data in the literature. In the construction of TLR clustering, we also utilized the structure of the helical assembly of the myddosome complex that is resolved by x-ray crystallography^13^. In addition, oligomerization modes of proteins are also taken into consideration. For instance, TRAF3 and TRAF6 proteins perform their functions by forming homo- or hetero-trimers.

### Mapping Oncogenic Mutations onto the Protein-Protein Interfaces and *in silico* Mutagenesis

Mutations of the proteins in the TLR network are obtained from cBioPortal for Cancer Genomics (The Cancer Genome Atlas, TCGA)^57^. We map oncogenic mutations to protein surfaces and select the ones that fall into the interface region. Interfaces or binding surfaces of the modeled protein-protein complexes are identified by the HotPoint server^44^. We perform *in silico* mutagenesis by using the FoldX plugin for the YASARA molecular viewer^60^ and re-run PRISM with the mutant structures to observe the effects of the mutations on the interactions. We minimized the energies of proteins before and after mutagenesis.

## Additional Information

**How to cite this article**: Guven-Maiorov, E. *et al.* The Architecture of the TIR Domain Signalosome in the Toll-like Receptor-4 Signaling Pathway. *Sci. Rep.* **5**, 13128; doi: 10.1038/srep13128 (2015).

## Supplementary Material

Supplementary Information
