# Supplementary material for: The Architecture of the TIR Domain Signalosome in the Toll-like Receptor-4 Signaling Pathway
Source: Sci Rep. 2015 Aug 21;5:13128. doi: 10.1038/srep13128 (PMC4544004; doi:10.1038/srep13128)
Supplement: Supplementary Information [file srep13128-s1.pdf]

SUPPORTING INFORMATION

The Architecture of the TIR Domain Signalosome in the Toll-like Receptor-4 Signaling Pathway

Emine Guven-Maiorov[1], Ozlem Keskin[1], Attila Gursoy[2*], Carter VanWaes[3], Zhong Chen[3], Chung-Jung Tsai[4], Ruth Nussinov[4,5*]

SI FIGURES

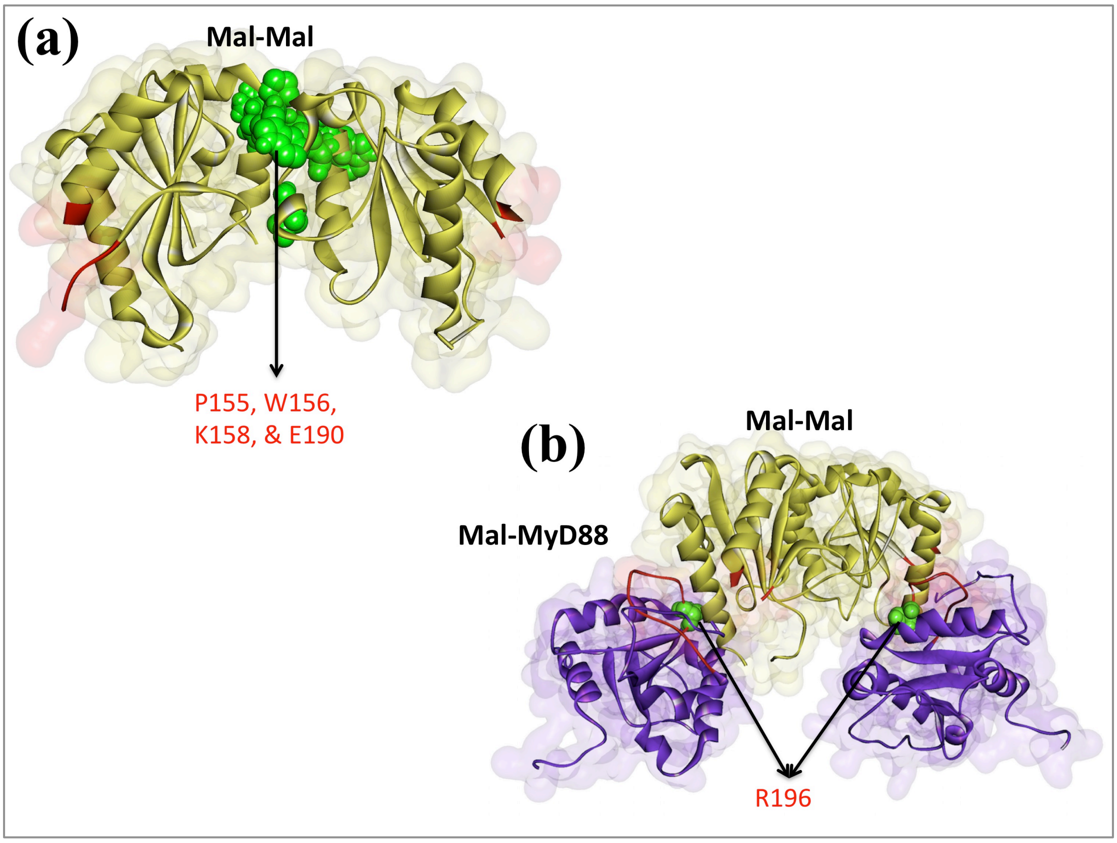

Figure S1, related to Figure 4: Interaction models of Mal-Mal, and Mal-MyD88. **(a)** Mal-homodimer model has the proposed interface residues (P155, W156, K158, and E190) (green spheres) [1, 2] at the correct site. Red-labeled regions are the residues of the AB-loop, the rest of the AB-loop residues (21 residues) are missing in the crystal

structure. **(b)** Interaction of two MyD88 molecules with a Mal-homodimer. R196 residue of MyD88 was previously suggested to be at the interface[2, 3] and is represented as green spheres in the figure. We obtained this complex by superimposing binary interaction models of Mal-Mal and Mal-MyD88.

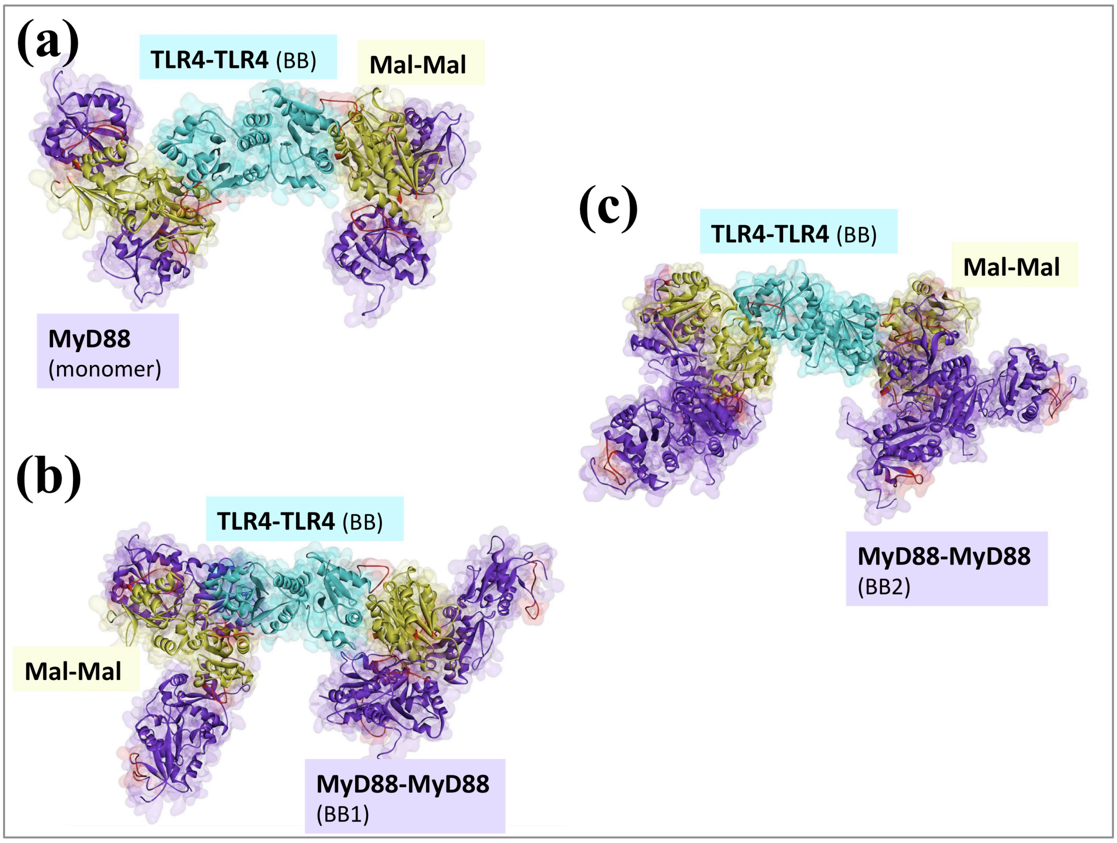

Figure S2, related to Figure 5: TIR-domain signalosomes with monomeric **(a)** and dimeric MyD88 molecules **(b & c)** for the BB TLR4 dimer. Oligomerization models of the large signalosome complex with two different back-to-back MyD88 dimers (BB1 and BB2). The TIR-domain signalosomes comprise a dimer of TLR4 interacting with two Mal-dimers, and four MyD88 dimers. We obtained this figure by superimposition of the binary interactions of TLR4-TLR4, TLR4-Mal, Mal-Mal, Mal-MyD88, and MyD88-MyD88.

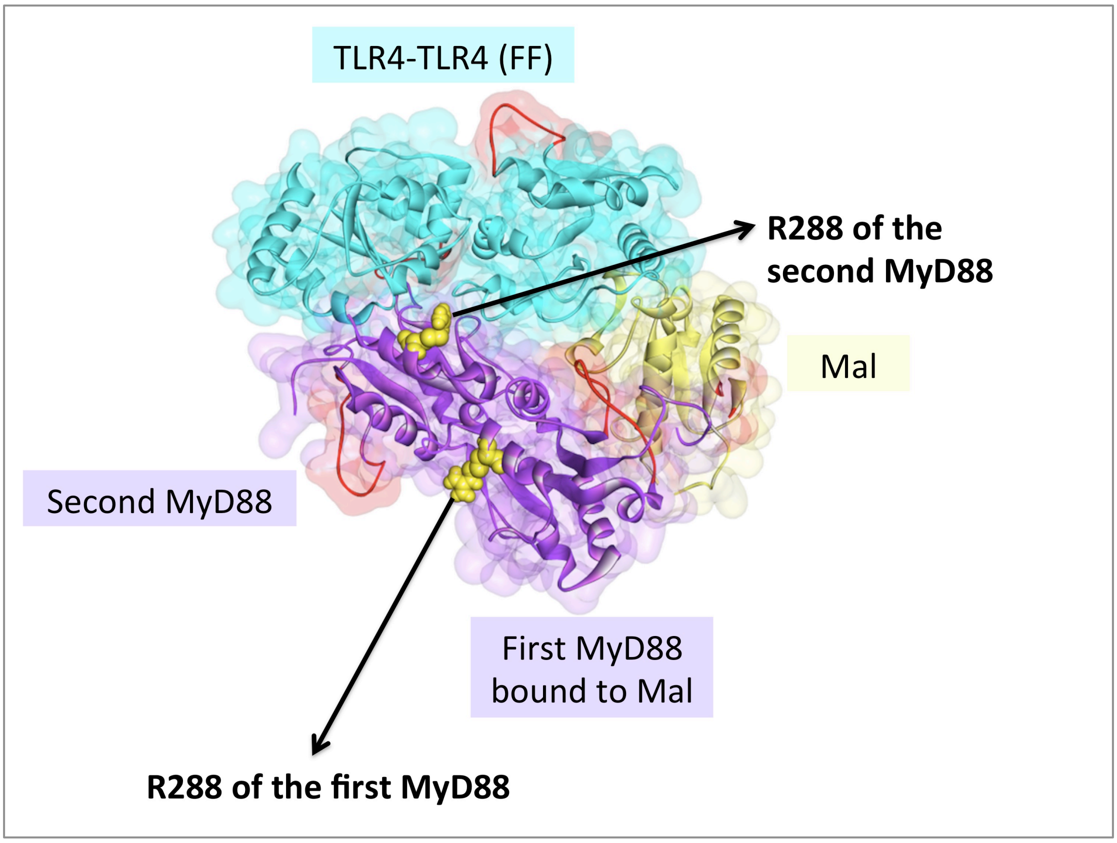

Figure S3, related to Figure 5: TLR4 dimer recruits Mal dimer, which in turn recruits MyD88-dimer (For simplicity only one Mal monomer is shown here). It has been suggested that MyD88-dimers have higher affinity to TLR4. The architecture we obtained supports this: when MyD88 dimerizes, one of the MyS88 monomers interacts with Mal, and the other is in contact with TLR4 itself. R196 and R288 of MyD88 have been shown to be at the Mal-MyD88 interface. Although R196 was at the Mal-MyD88 interface (Figure S1a), R288 is found at the TLR4-MyD88 interface.

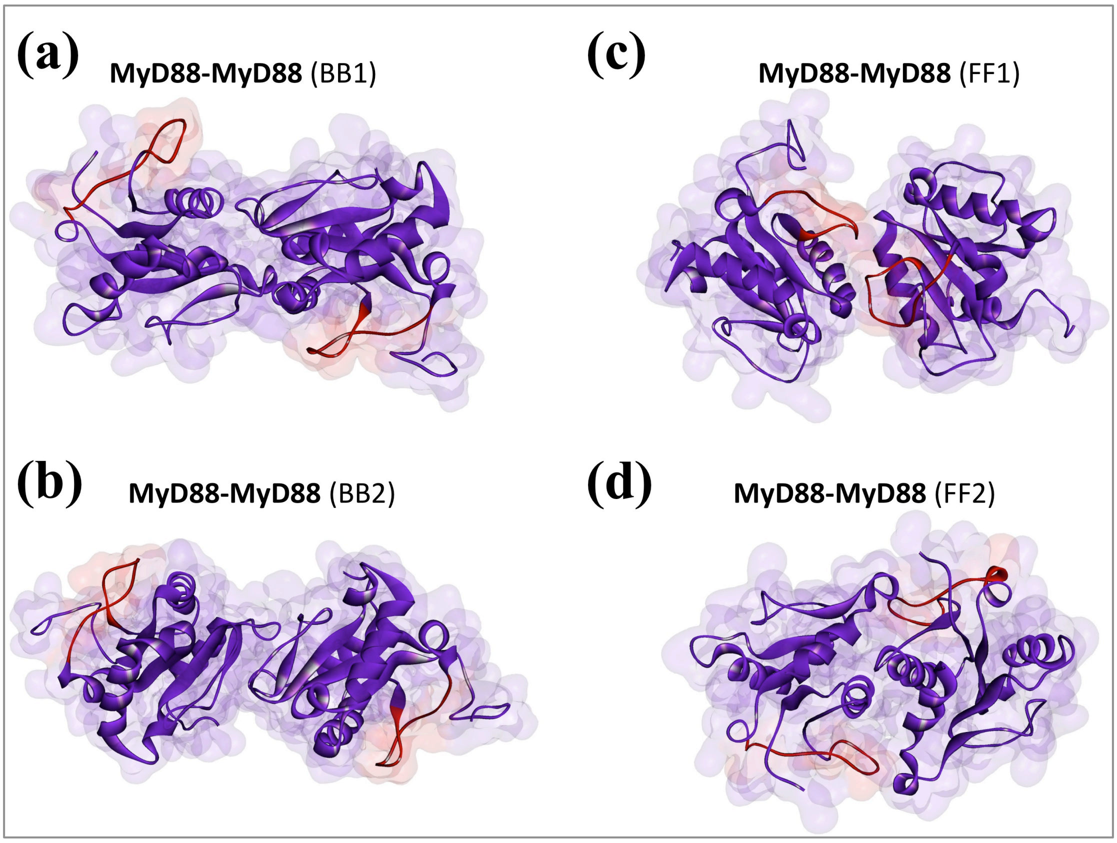

Figure S4, related to Figure 5: Four MyD88- dimer models: **(a & b)** back-to-back dimer models (BB1 and B2) and **(c & d)** face-to-face dimer models (FF1 and FF2). Only BB1 and BB2 are feasible with our TIR-domain signalosome models.

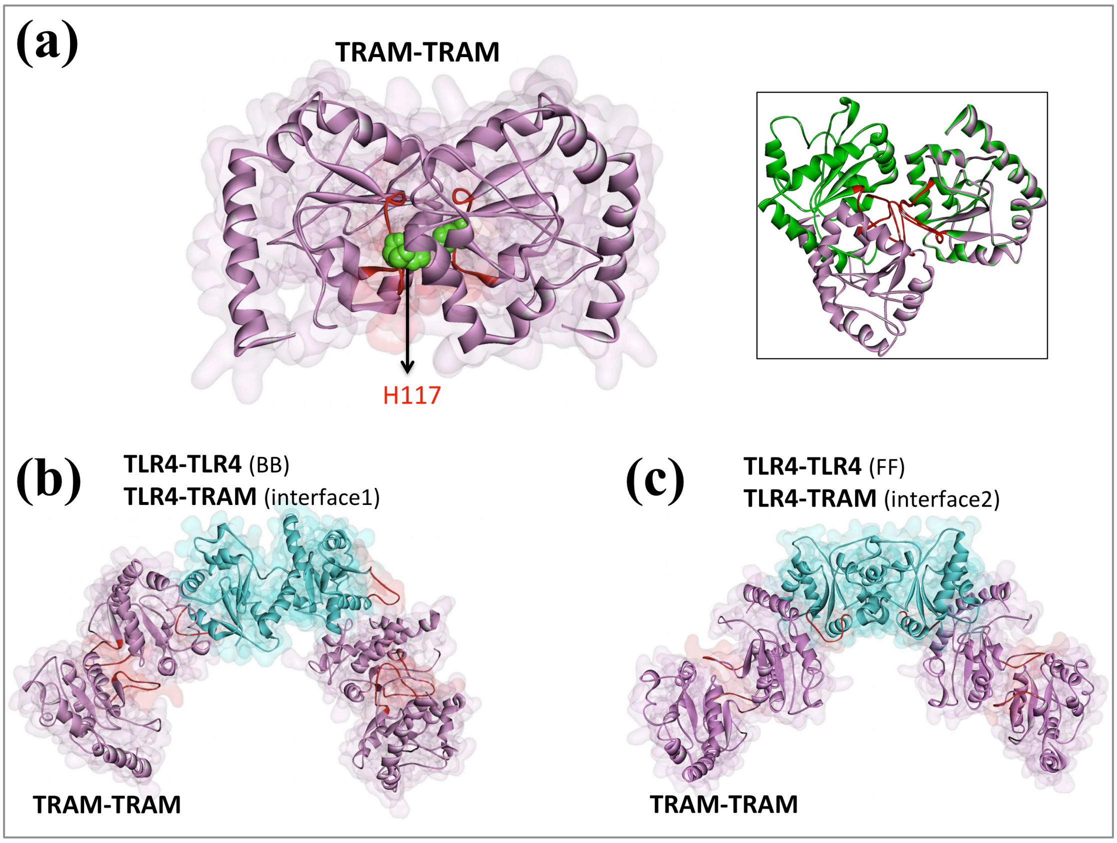

Figure S5, related to Figure 6: A detailed view of TRAM-homodimer model and its interaction with the two opposite TLR4 dimer models. **(a)** TRAM homodimer model, with BB-loops facing each other. Small box at the top right corner shows the superimposition of TRAM-homodimer model obtained by PRISM with TRAM-homodimer obtained by superimposition with TLR10 homodimer in the crystal structure (2j67_AB). Pink monomers are TRAM TIR domains; red-labeled regions are BB-loops of TRAMs; green spheres show the H117 residue of TRAM, which is suggested to be at the interface [4]. **(b & c)** TLR4-TRAM interaction model with BB and FF TLR4-homodimer models, respectively.

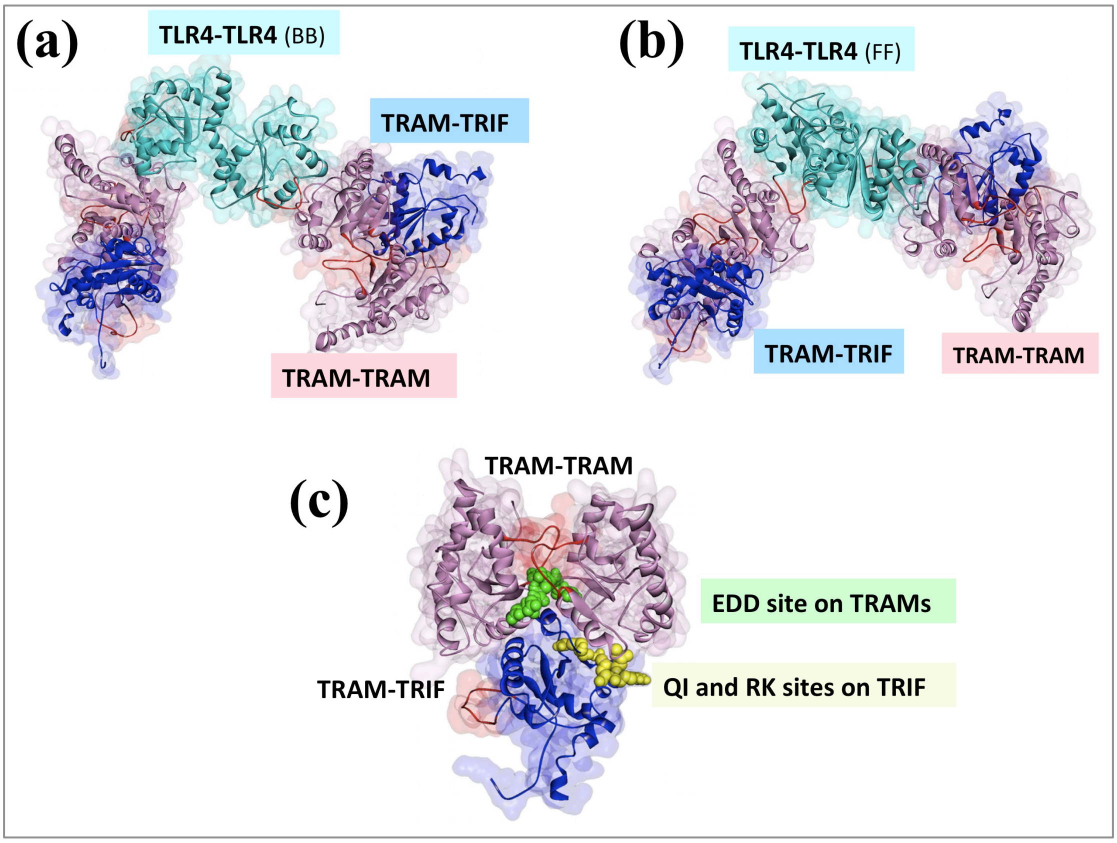

Figure S6, related to Figure 6: TRAM-TRIF interaction and TRIF-dependent TIR-domain signalosome for BB TLR4 dimer. **(a)** TRIF-dependent TIR-domain signalosome model of BB TLR4-dimer, which involves TRAM-dimer and TRIF-monomer. **(b)** TRIF-dependent TIR-domain signalosome model for FF TLR4-dimer. **(c)** TRAM-TRIF interaction model. The residues that are proposed to be at the TRAM-TRIF interface include Q512, I519 (QI-site), R522, K523 (RK-site) of TRIF and T155, S156 (TS-site), E87, D88, D89 (EDD-site) of TRAM [4]. The TRAM-TRIF interaction model has QI-, RK- and EDD-sites at the interface, but not the TS-site. Yellow spheres are residues Q512, I519 (QI site), and R522, K523 (RK-site) of TRIF; green spheres are residues E87, D88, D89 (EDD-site) of TRAM proteins in the homodimer. These residues are suggested to be at the interface by a recent study [4], which is in accordance with our model. This TRAM-TRAM-TRIF interaction model is possible with both FF and BB TLR4 homodimer models. Cyan protein is TLR4;

yellow is Mal; purple is MyD88; pink is TRAM; blue is TRIF; and red-labeled regions are BB-loops.

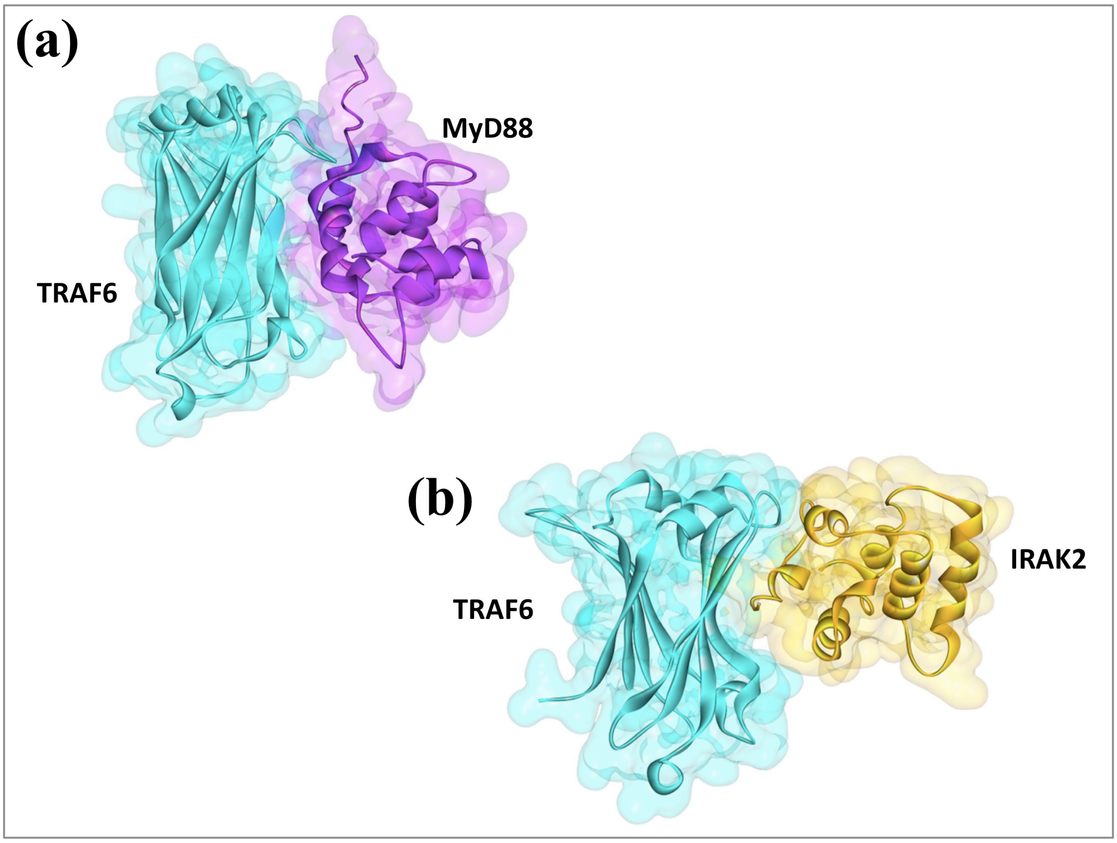

Figure S7, related to Figure 7: Interaction models of TRAF6 with monomeric MyD88 and IRAK2.

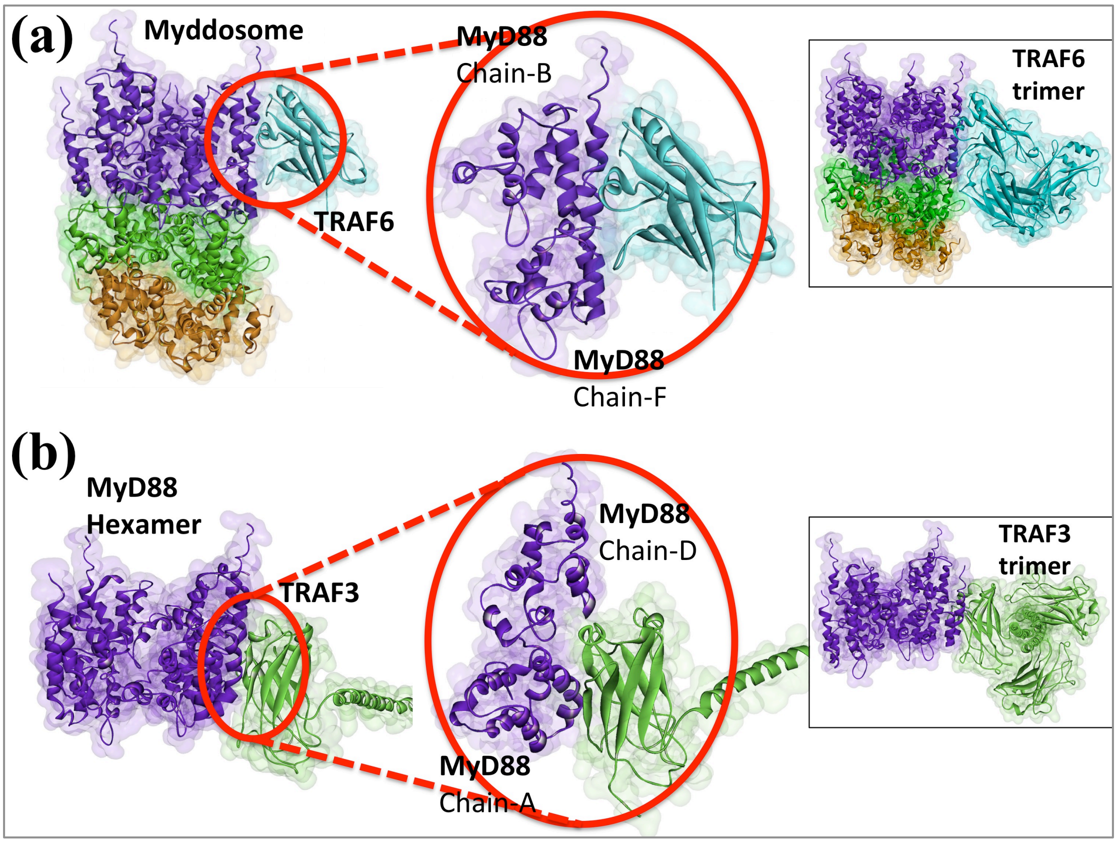

Figure S8, related to Figure 7: Interaction models of MyD88 with TRAF6 and TRAF3. **(a)** Myddosome-TRAF6 interaction model. TRAF-C domain of TRAF6 prefers to bind to MyD88 when the whole myddosome structure is given as a target. The interface is very similar to the concave TRAF binding site with peptides as observed in TRAF6-CD40 (1lb6.pdb) [5], TRAF2-TRADD (1f3v.pdb) [6], and TRAF2-OX40 (1d0a.pdb) [7]. TRAF6 is in contact with two monomers of MyD88 (two layers of MyD88) in the myddosome assembly. In order to execute its function, TRAF6 needs to trimerize and TRAF6 trimerization is also possible with our model. **(b)** Interaction model of TRAF3 with hexameric MyD88 in the myddosome. Like TRAF6, TRAF-C region of TRAF3 is in contact with two MyD88 monomers (two layers of MyD88). The interface is very similar to the concave TRAF binding site observed in other interactions, such as TRAF3-CD40 (1fll.pdb) [8], TRAF3-BAFFR (2gkw.pdb) [9], TRAF3-LMP1 (1zms.pdb) [10], and TRAF3-Cardif (4ghu.pdb) [11].

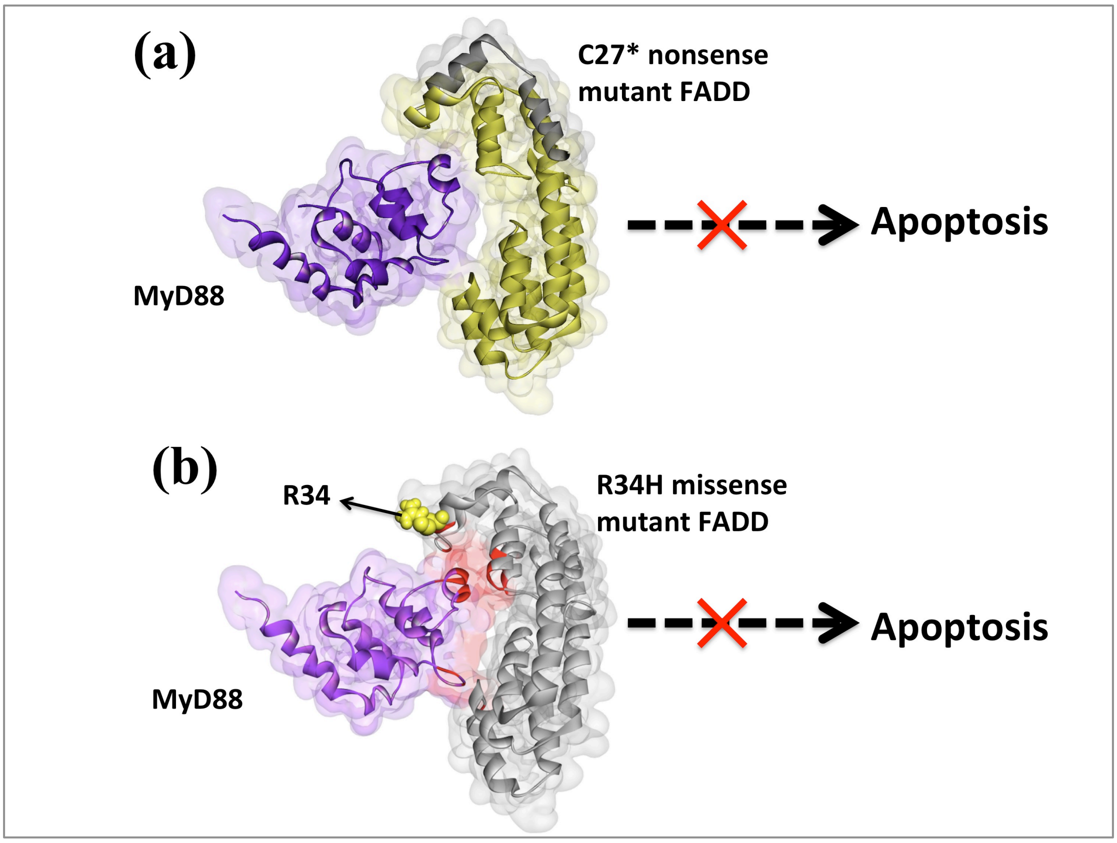

Figure S9, related to Figure 7: A nonsense and a missense mutation on FADD prevents FADD-MyD88 interaction. **(a)** C27* nonsense mutation on FADD abolishes its interaction with MyD88, since large portion of the interface would be missing in the mutant FADD (yellow-labeled part on FADD). **(b)** R34H missense mutation on FADD falls just next to interface region and abrogates FADD-MyD88 interaction. Red-labeled residues are the interface residues and Arg-34 residue is labeld with yellow.

SI TABLES

Table S1: PRISM predictions used to construct structural TLR network.

PPI	Protein1	Protein2	Template Interface	Interaction Energy
**TLR4-TLR4** (BB)	Homology model	Homology model	2g37AB	-31.45
**TLR4-TLR4** (FF)	Homology model	Homology model	1z41AB	-17.68
**TLR4-TLR4** (FF2)	Homology model	Homology model	1ufoCD	-22.98
**TLR4-Mal** (interface1)	Homology model	3ub2A	1c0gSA	-15.41
**TLR4-Mal** (interface2)	Homology model	4lqdA	1pzmAB	-25.4
**Mal-Mal**	3ub2A	3ub2A	3urrAB	-14.46
**Mal-MyD88**	4lqdA	2js7A	1mo1AB	-27.07
**MyD88-MyD88** (BB1)	2z5vA	2z5vA	3lyhAB	-22.52
**MyD88-MyD88** (BB2)	2js7A	2js7A	1a96AB	-16.8
**MyD88-MyD88** (FF1)	2js7A	2js7A	1bjfAB	-35.74
**MyD88-MyD88** (FF2)	2z5vA	2z5vA	1gylAB	-30.96

**TLR4-TRAM (interface1)**	Homology model	2m1wA	3gmgAB	-15.94
**TLR4-TRAM (interface2)**	Homology model	2m1wA	3o2qDE	-15.24
**TRAM-TRAM**	2m1wA	2m1wA	1pzmAB	-24.63
**TRAM-TRIF**	2m1wA	2m1xA	3urrAB	-19.11
**MyD88-TRAF6**	3mop	1lb5A	3lfrAB	-11.63
**MyD88-TRAF3**	3mopABCDEF	1fllA	2oh1CD	-16.77
**MyD88-FADD**	3mopA	2gf5A	1mu4AB	-18.33

SI REFERENCES

1. Bovijn, C. et al. Identification of binding sites for myeloid differentiation primary response gene 88 (MyD88) and Toll-like receptor 4 in MyD88 adapter-like (Mal). *J Biol Chem* **288**, 12054-66 (2013).

2. Valkov, E. et al. Crystal structure of Toll-like receptor adaptor MAL/TIRAP reveals the molecular basis for signal transduction and disease protection. *Proc Natl Acad Sci U S A* **108**, 14879-84 (2011).

3. Ohnishi, H. et al. Structural basis for the multiple interactions of the MyD88 TIR domain in TLR4 signaling. *Proc Natl Acad Sci U S A* **106**, 10260-5 (2009).

4. Enokizono, Y. et al. Structures and interface mapping of the TIR domain-containing adaptor molecules involved in interferon signaling. *Proc Natl Acad Sci U S A* **110**, 19908-13 (2013).

5. Ye, H. et al. Distinct molecular mechanism for initiating TRAF6 signalling. *Nature* **418**, 443-7 (2002).

6. Park, Y.C. et al. A novel mechanism of TRAF signaling revealed by structural and functional analyses of the TRADD-TRAF2 interaction. *Cell* **101**, 777-87 (2000).

7. Ye, H., Park, Y.C., Kreishman, M., Kieff, E. & Wu, H. The structural basis for the recognition of diverse receptor sequences by TRAF2. *Mol Cell* **4**, 321-30 (1999).

8. Ni, C.Z. et al. Molecular basis for CD40 signaling mediated by TRAF3. *Proc Natl Acad Sci U S A* **97**, 10395-9 (2000).

9. Ni, C.Z. et al. Key molecular contacts promote recognition of the BAFF receptor by TNF receptor-associated factor 3: implications for intracellular signaling regulation. *J Immunol* **173**, 7394-400 (2004).

10. Wu, S. et al. LMP1 protein from the Epstein-Barr virus is a structural CD40 decoy in B lymphocytes for binding to TRAF3. *J Biol Chem* **280**, 33620-6 (2005).

11. Zhang, P. et al. Single amino acid substitutions confer the antiviral activity of the TRAF3 adaptor protein onto TRAF5. *Sci Signal* **5**, ra81 (2012).
